# EFFECTS OF ACUTE EXERCISE AND A DUAL COGNITIVE TASK ON A NOVEL CIRCULATING BIOMARKER

**DOI:** 10.1093/geroni/igae098.3960

**Published:** 2024-12-31

**Authors:** Madison Croucher, Jennifer R McCall, Kathryn Sausman, Lidio L de Albuquerque, Tamatha Arms, Matthew Peterson

**Affiliations:** University of North Carolina Wilmington, Wilmington, North Carolina, United States; University of North Carolina Wilmington, Wilmington, North Carolina, United States; University of North Carolina Wilmington, Wilmington, North Carolina, United States; University of North Carolina Wilmington, Wilmington, North Carolina, United States; University of North Carolina Wilmington, Wilmington, North Carolina, United States; University of North Carolina Wilmington, Wilmington, North Carolina, United States

## Abstract

This study reports on the effect of aerobic exercise, with or without a concurrent cognitive task on circulating levels of beta hydroxybutyric acid (b-HBA) and glucose in older adults with normal cognition.

**Methods:**

Two bouts of exercise one week apart were performed to examine pre-post b-HBA and glucose in, 1) 10-minutes of stationary bicycling, and 2), 10-minutes of stationary bicycling with a cognitive challenge. Variables included cognitive screening (MoCA), fasting blood samples, health status, physical performance (SPPB), hand function, and depressive symptoms.

**Results:**

N=10 female participants were 90% Caucasian, 71.6 +/-4.3 years old with 3.2 +/-2.1 comorbidities, normal cognitive functioning (MoCA=27 +/-1.5) and good physical performance (SPPB 11.1 +/-1.1). There was a clinically meaningful positive association between change in circulating concentrations of b-HBA and MoCA scores with the dual task condition (r=0.32). Glucose was utilized more in the exercise only task compared to the dual condition task (Visit 2 – Visit 1 difference: mean increase of 9.5% +/- 16.4% in pre-post % change), whereas b-HBA was depleted in the dual task condition (Visit 2-Visit 1 difference: mean decrease of 17.0% +/- 33.4% in pre-post % change). Both b-HBA and glucose concentrations changes had moderate effect sizes (Cohen’s D=0.51 and 0.57, respectively). Conclusions: This study provides proof of concept for differing energy substrate utilization, when the brain is tasked with a cognitive challenge added to an already moderately intense whole-body cycling exercise. Future studies will objectively quantify energy substrate utilization in the brain using this experimental design and imaging studies.

